# The player–pony dyad in Polo: lessons from other sports and future directions

**DOI:** 10.1093/af/vfac003

**Published:** 2022-06-14

**Authors:** Russ Best

**Affiliations:** Centre for Sport Science & Human Performance, Waikato Institute of Technology, Hamilton, New Zealand; Kihikihi Polo Club, Kihikihi, New Zealand; Tiger Polo Academy, Mystery Creek, Hamilton, New Zealand

**Keywords:** equestrian, equine, human–horse interaction, interaction, performance analysis, polo

**Implications**

• The interaction between the horse–athlete dyad is fundamental to successful performance in equestrian sport.

• Quantification of the dyad is of interest to a range of sports science disciplines and may be achieved objectively or subjectively.

• Polo presents an ideal model to assess the horse–rider dyad due to the high volume of interactions and a high degree of repeatability.

## Introduction 

The horse–human dyad is a requisite foundation for all equestrian sports. The interaction between humans and horses has been studied using a suite of methodologies, seemingly incorporating the extremes of quantitative (e.g., human and equine biomechanists, veterinarians) and qualitative approaches (e.g., equine behavior, sports psychologists). All approaches have validity, and in practice, a middle-ground approach is likely warranted. The incorporation of athletes and coaches into the research process is strongly encouraged to enable more meaningful data to be obtained and transferred into practice. This is particularly important in an equestrian sport where the human athlete may employ a coach or mentor (Lamperd et al., 2016), but is also effectively coaching the equine athlete(s) through their riding and other schooling. Data, therefore, should be used to objectively inform positive interactions for both athlete and horse, supporting qualitative factors, and not simply gathered for its own sake.

A further rationale that may predicate both quantitative and qualitative approaches is that much of horse riding is considered tacit knowledge. That is to say, riding is a skill that is acquired experientially through exposure to various contexts and may not always be easily communicated (Blokhuis and Lundgren, 2017). Developing the skill set to educate and improve a wide range of horses (age, breeding, sex, etc.), and concomitantly oneself as a rider, is considered a requisite to elite equestrian performance and may manifest acutely or longitudinally (Lamperd et al., 2016). This is reflected in both human and equine elements of the horse–rider dyad, with behavioral ethograms (Dyson et al., 2020) and measures of heart rate (**HR**) or HR variability (Visser et al., 2002, Scopa et al., 2020) used as conduits for verbal communication in equids, whereas riders may be able to verbalize a horse’s behavioral tendencies. A third language describes the mounted human–horse interaction (Brandt, 2004), this is non-native to either species, yet directly influences equine and human performance, by manipulating pressure signals at the points of human–horse interaction. This will vary between disciplines and depend heavily on equipment used but typically include pressure through the saddle, the side of the horse and rider’s legs, and the reins, bit, and rider’s hands (Brandt, 2004; Blokhuis and Lundgren, 2017). 

This perspective will argue that Polo is the ideal equestrian sport in which to study the horse–athlete dyad, outline appropriate technologies for dyad assessment, and that there is much to learn from the wider sporting community on assessment and evidence-informed practice. Elite equestrian athletes have previously reported the benefits of having “eyes on the ground,” coaches who provide objective interpretations of their performance (Lamperd et al., 2016). The incorporation of technologies to assess or inform performance allows for an objective quantification of performance signals, across multiple systems, and removes bias due to anthropomorphism or emotiveness (Blokhuis and Lundgren, 2017), at the level of the athlete, horse. or dyad depending on the technologies employed. Data can then be used to reinforce tacit knowledge (e.g., how adopting a different seat alters a horse’s gait), changes in feel within or between horses (e.g., horses of differing competitive experience), or to track trends over competitive seasons (e.g., horse and athlete cardiovascular fitness). Please note that throughout this perspective, the terms player and pony are preferred when discussing Polo but are referred to as athlete and horse for other disciplines.

## Polo as a Model of Interaction

Polo presents a seemingly ideal model to study the horse–human (player–pony) dyad, as players are required to undertake multiple interactions per game, often per chukka (period of play), that is, players ride at least one horse per chukka and horses are not permitted to play consecutive chukkas. Players typically maintain a consistent playing order of their horses (referred to as a string) through a tournament, enhancing possible experimental repeatability/reliability further. This volume and repeatability of interactions are not apparent in any other equestrian discipline, so that practitioners can make relatively consistent observations on the same player and the same or similar string of horses across a season and sometimes at differing levels of play (e.g., Best and Standing, 2019a, 2019b). Likewise, tack is often interchanged between horses, and while this provides a more consistent platform for the athlete, every effort should be made to ensure that horse comfort and welfare are not adversely impacted. Objective assessment of key points of interaction and concomitant horse behaviors may support horse welfare in Polo by ensuring correct saddle fitting and bitting.

The reverse is true for eventing, where a single rider contests three disciplines on the same horse. One may see as great a degree of variability within the rider between disciplines as within the horse, depending upon event preference, level of expertise, and level of competition, adding *noise* to any data collected but potentially strengthening the quantitative understanding of an athlete’s riding “signature” and how this is expressed in relation to event demands.

A further idiosyncrasy of Polo is the handicapping system, where players are rated from −2 to 10 goals, and the cumulative handicap of the four players on a team dictates the level of play (e.g., 22 goals—the standard for “high goal” Polo in the United Kingdom). Anecdotally, players report that 70% to80% of the success of their game is attributed to their string of ponies. Quantifying this would require detailed analysis of player and pony factors, such as performance analysis of shots performed and their success rates (Best and Standing, 2019c), physiological responses of both player and ponies to the work performed (Best and Standing, 2021), and how this varies between each unique player–pony interaction while accounting for player handicap and level of play.

This is not impossible but presents a paradox. A lower handicapped player may be a less effective rider and thus is more likely to require “made” horses to afford opportunity for athlete skill development. A higher handicapped player may be a more effective rider but requires better and more horses to sustain the increased pace, high-intensity movements, and technical nature of high-goal Polo (Best and Standing, 2019a). The suggested contribution of horses to one’s game quickly appears reasonable, if not conservative. Ultimately, players, regardless of level, require positive interactions with their ponies—the measurement of which can be altered to suit player deficiencies or inform research questions.

It is important to note that like other equestrian pursuits there is a business aspect to elite-level Polo players’ performance. Professional equestrians are required to ensure that every horse performs at its best, under its current developmental constraints (Lamperd et al., 2016). Horses can be developed and sold to ensure sustainability within the sport, but, unlike other equestrians, Polo players must play to their handicap and success is judged on a win/loss outcome (Best and Standing, 2019b, 2019c), as opposed to rankings across multiple competitive rounds. This may impact the potential for the formation of long-term player–pony partnerships, as players may sell a horse either to a higher handicapped player for further development or prestige or to lower-ability players if a horse’s developmental potential has been realized or it does not possess the aptitude for higher levels of play. This pressure is not unique to Polo or equestrian sports (e.g., racing, dressage) but is not widely considered as a confounding variable in (longitudinal) sports science research that implements some of the technologies included in this commentary.

## Technologies: What Do Interactions Tell Us?

When assessing the components of the player–pony dyad, the following technologies are available and are currently employed either in Polo and/or in other equestrian disciplines:

• Global positioning systems (**GPS**) can quantify the external load (speed, distance, and distance rate) experienced by a horse or a player–pony dyad. Polo is now quantified in some detail, with an appreciation for different levels of play and differences between Open and Women’s Polo (Best and Standing, 2019a). GPS units tend to be reliable (Best and Standing, 2019d) and are easily paired with other metrics to assess the dyad more meaningfully, thereby promoting a rounded approach to pony and player management.

• HR is commonly assessed in both humans and equines, with research in Polo confirming its status as a demanding sport for both players and ponies (Marlin and Allen, 1999; Best and Standing, 2021). Further investigation is required, however, especially concerning within- and between-species comparisons. This has been done to some extent in equitation science, where HR responses to unfamiliar tasks or humans may be examined (Visser et al., 2002; Scopa et al., 2020), but an assessment of dyad HR responses that are correlated to external load would be highly valued.

• Blood lactate (**BLa**) monitoring is a routine protocol in exercise laboratories, and with portable handheld devices, it is easily transferred to field-based testing sessions. Despite this convenience, and the high reliability and validity of devices and protocols used, BLa testing within equestrian settings is predominantly used to assess the intensity of work performed by equine rather than human equestrian athletes, especially in Polo (Ferraz et al., 2010; Gondin et al., 2013; Noleto et al., 2016). In other disciplines, BLa production has been reported to be ~≥80% of lab-derived maxima (Hyttinen et al., 2020), indicating a marked glycolytic contribution to equestrian exercise; BLa levels also appear to be lower in more experienced riders, suggesting either an increased aerobic contribution or increased BLa utilization for energy production, possibly due to increased training frequency. BLa values may be higher in Polo, due to increased upper body involvement, through swinging of the mallet and impacts sustained when riding-off other players, but this hypothesis requires confirmation.

• Inertial measurement units are arguably the most labor-intensive technology used within equitation research. Either units can be used in isolation, to provide an estimated global load experienced by an athlete (Burla et al., 2014), or multiple units can be used synchronously to assess joint movement and/or loading (Bastiaansen et al., 2020). The main limitation here is interpreting how such forces transfer to or from the pony, depending upon the action in question, and doing so in a manner that minimizes “false positives.’ In our experience, even normal riding actions, such as a player slowing a horse using their seat, may require the rider to absorb considerable force and may trigger an arbitrary impact threshold within the device, depending upon device placement.

• Pressure monitoring has also been employed to assess targeted points of contact between rider and horse, typically across a range of gaits or athlete skill levels. These include bridles and bits, reins, saddle, and stirrup pressures (van Beek et al., 2012; Nicol et al., 2014), allowing the assessment of how an interaction occurs (severity, time, etc.), but the technology required may not always transfer well into a competitive setting, thereby potentially limiting ecological validity.

**Figure 1.**
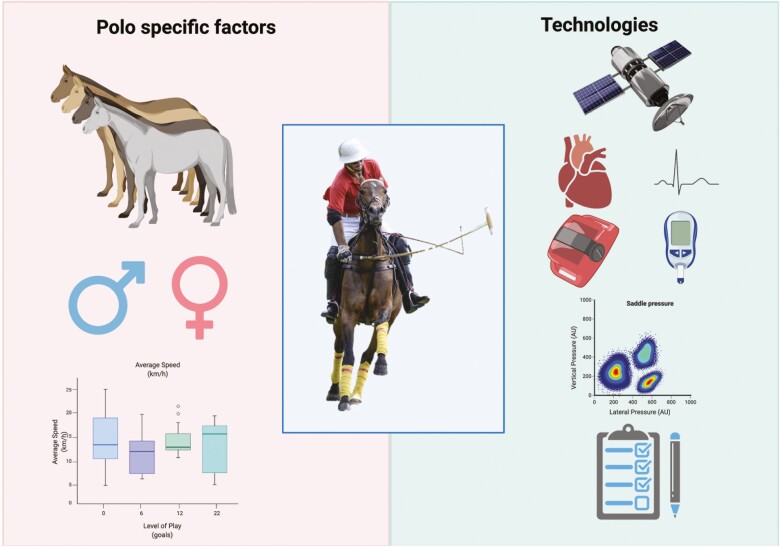
Polo-specific factors and technologies used to assess the player–pony dyad. Polo-specific factors (Left) include (from the top to bottom) within- and between-horse variability, Open or Women’s Polo, and level of play. Technologies (Right) include (from the top to bottom) GPS, HR and HR variability, inertial measurement units, BLa meters, pressure sensors, and subjective ratings. This figure is made with Biorender.com.

Given the importance of tacit knowledge and inter-species communication in equestrian sports, subjective assessments of interactions are also encouraged, especially in conjunction with objective measures; this work is in its infancy, but a lot can be gleaned from sport science (see later). The potential difference in reliability and validity between commercially available and research-grade devices is a consideration of scientists encouraging the adoption of technologies in equestrian training and competitive environments. Devices that are consistently inconsistent (reliable but may lack validity) are favored over those that are inconsistently inconsistent (neither reliable nor valid), as tracking changes as a result of an intervention or trends over time may still be possible through corrective equations (Figure 1). 

## What Can We Learn from Other Team Sports?

Based on the above, there is an emerging need for standardized testing procedures in equestrian sport as well as exposing riders to different athlete–horse pairings to maximize tacit learning opportunities. This also facilitates the assessment of intra-rider/inter-equine variability or vice-versa. Any technology not only should display an appropriate level of sensitivity but should also be applied specifically to the challenges of a discipline and positioned to answer the research question, while minimizing the risk of injury to the player–pony dyad and maximizing horse welfare.

Team sports commonly gather subjective ratings of perceived exertion (RPE), post-training, or competition. While RPE assessment in equestrian athletes would add value, athletes may also consider quantifying their horses’ performance using a matched scale. This could then be corroborated and correlated to some of the metrics mentioned earlier (e.g., GPS and HR; Scherr et al., 2013). Likewise, data from technologies and subjective ratings of player or pony performance can be paired with external input from those who are able to interpret an athlete’s performance, critically and objectively (Lamperd et al., 2016). The manner in which this information is fed back to the athlete, and how this is implemented across an athlete’s career, is likely as pivotal to successful intervention as to how data from technologies are initially parsed to meaningfully represent athletic performance signals for the pony, player, or dyad. Importantly, all gathered data should inform practice or at least serve as an educational tool for the development of the player, so that practitioners are encouraged to consider how these data transfer from device to action.

## Summary

Practitioners are well served by gathering (quantitative) data that are specific to an equestrian discipline and its constraints. Polo is seemingly ideal, as it requires players to undertake multiple player–pony interactions per game and even per chukka. This is challenging, so where technologies are employed, ensuring that sufficient data are gathered across a range of contexts should be prioritized (e.g., Best and Standing, 2019a). Second to this, these data should be reliable and valid so that one can be confident in the obtained measures and changes therein. How these data relate to performance outcomes can then be considered (e.g., Best and Standing, 2019b). Data from technologies, therefore, support the athlete and the horse and allow for a rigorous approach to the advancement of equestrian performance through an integrated and systematic approach. Finally, reflecting upon these data with coaches, players, and other practitioners allows for better data to be collected in the future, ensuring that measurements are meaningfully communicated and implemented.

## About the Author

**Russ Best** is a Principal Academic Staff Member at the Waikato Institute of Technology’s Centre for Sport Science & Human Performance, where he is also Research Leader and heads the center’s ergogenic strategies and equestrian performance research groups. His current research interests include quantifying the physiological and performance demands of Polo and strategies to augment exercise performance and health in the heat. Outside of academia, he is an amateur Polo player at the Kihikihi Polo Club, former long-distance runner, and husband and father.
